# Predictors for new-onset conduction block in patients with pure native aortic regurgitation after transcatheter aortic valve replacement with a new-generation self-expanding valve (VitaFlow Liberty^™^): a retrospective cohort study

**DOI:** 10.1186/s12872-024-03735-z

**Published:** 2024-01-28

**Authors:** Xuan Zhang, Cheng Liang, Lintao Zha, Quan Zuo, Guobing Hu, Jie Ding, Shengxing Tang

**Affiliations:** 1 Department of Cardiology, The First Affiliated Hospital (Yijishan Hospital) of Wannan Medical College, No. 2, Zheshan West Road, Jinghu District, Wuhu City, Anhui Province China; 2 Department of Ultrasound Medicine, The First Affiliated Hospital (Yijishan Hospital) of Wannan Medical College, Wuhu, Anhui China

**Keywords:** Transcatheter aortic valve replacement, Permanent pacemaker implantation, Pure native aortic regurgitation, Atrioventricular block, Complete left bundle branch block, Complete right bundle branch block

## Abstract

**Background:**

New-generation self-expanding valves can improve the success rate of transcatheter aortic valve replacement (TAVR) for severe pure native aortic regurgitation (PNAR). However, predictors of new-onset conduction block post-TAVR using new-generation self-expanding valves in patients with PNAR remain to be established. Therefore, this study aimed to identify predictors of new-onset conduction block post-TAVR using new-generation self-expanding valves (VitaFlow Liberty™) in patients with PNAR.

**Methods:**

In this retrospective cohort study, patients were categorized into pacemaker and non-pacemaker groups based on their need for new postoperative permanent pacemaker implantation (PPI). Based on the postoperative presence of either new-onset complete left bundle branch block (cLBBB) or high-grade atrioventricular block (AVB), patients were further classified into conduction disorder and non-conduction disorder groups. Laboratory, echocardiographic, computed tomography, preoperative and postoperative electrocardiography, and procedural and clinical data were collected immediately after TAVR and during hospitalization and compared between the groups. Multivariate logistic regression analysis was performed incorporating the significant variables from the univariate analyses.

**Results:**

This study examined 68 consecutive patients with severe PNAR who underwent TAVR. In 20 patients, a permanent pacemaker was fitted postoperatively. Multivariate logistic regression analysis revealed an association between the need for postoperative PPI and preoperative complete right bundle branch block (cRBBB) or first-degree AVB, as well as a non-tubular left ventricular outflow tract (LVOT). In addition, valve implantation depth and angle of aortic root were independent predictors of new-onset cLBBB or high-grade AVB developing post-TAVR. The predictive value of valve implantation depth and angle of aortic root was further supported by receiver operating characteristic curve analysis results.

**Conclusions:**

In patients with PNAR undergoing TAVR using self-expanding valves, preoperative cRBBB or first-degree AVB and a non-tubular LVOT were indicators of a higher likelihood of PPI requirement. Moreover, deeper valve implantation depth and greater angle of aortic root may be independent risk factors for new-onset cLBBB or high-grade AVB post-TAVR. Valve implantation depth and angle of aortic root values may be used to predict the possibility of new cLBBB or high-grade AVB post-TAVR.

**Supplementary Information:**

The online version contains supplementary material available at 10.1186/s12872-024-03735-z.

## Background

Transcatheter aortic valve replacement (TAVR) has been preferred as the main treatment method for severe aortic stenosis (AS) owing to its safety and effectiveness [1–6]. With growing expertise and significant clinical effectiveness in treating severe AS using TAVR, along with continuous technical improvements and advancements in device development [7–9], the method is now widely employed in patients with anatomical diversity, including those with bicuspid aortic valves and failing surgical bioprosthetic valves [4, 5, 7, 10]. In recent years, researchers have been exploring the feasibility of TAVR for off-label indications, such as pure native aortic regurgitation (PNAR) [11, 12].

The accumulation of TAVR experience, coupled with improvements in technical parameters and valve devices, has contributed to the safety and feasibility of using TAVR in the treatment of PNAR [13, 14]. Compared with the first-generation valve devices, new-generation valve devices have been demonstrated to have better anchoring in the absence of significant aortic valve calcification in patients with PNAR [13–15]. Numerous studies have shown that new-generation self-expanding valves can improve the success rate of TAVR in patients with PNAR and reduce complications, including paravalvular leakage and more [13, 14, 16–19]. However, new-generation valves do not reduce the need for permanent pacemaker implantation (PPI) in patients with PNAR after TAVR [13, 14, 16–20].

PPI is commonly required after TAVR, with registry studies reporting the use of PPI in 7.5–27.3% in patients with PNAR after TAVR [13,16,19,21,22]. New conduction block after TAVR seriously affects the prognosis of patients with aortic valve disease. A meta-analysis has shown that PPI after TAVR markedly increases the rates of all-cause mortality and rehospitalization due to heart failure [23]. The presence of a new postoperative left bundle branch block (LBBB) is an independent predictor for New York Heart Association functional class III or IV after TAVR for PNAR [15]. Ananwattanasuk et al. revealed that high-load right ventricular pacing or the presence of permanent LBBB after TAVR significantly increased the risk of 1-year postoperative mortality and cardiomyopathy [24].

Data from our hospital revealed that patients with PNAR were more prone to developing new conduction blocks requiring PPI compared to patients with AS [25]. Evidently, there is an urgent need for further research to investigate the causes of these complications and to optimize the management and treatment of patients requiring PPI after TAVR. Therefore, our study aimed to determine the clinical predictors of both PPI and new-onset conduction block after TAVR in patients with PNAR using a new-generation self-expanding valve (VitaFlow Liberty™, MicroPort, Shanghai, China).

## Methods

### Participants

In this retrospective cohort study, electronic medical records were reviewed for all patients who underwent TAVR for severe PNAR at the Cardiac Center of the First Affiliated Hospital (Yijishan Hospital) of Wannan Medical College in Wuhu, China, from November 2021 to June 2023. Before performing TAVR for all patients, a multidisciplinary collaborative discussion involving cardiology, cardiac surgery, ultrasound medicine, anesthesiology, medical imaging, and digital subtraction angiography (DSA) experts was conducted and documented in patients’ records. All patients included in this study were considered to be at high risk for or to have contraindications for surgical aortic valve replacement. Patients with incomplete medical records were excluded from the study.

The study was approved by the Medical Ethics Committee of The First Affiliated Hospital (Yijishan Hospital) of Wannan Medical College and complied with the principles set forth by the Declaration of Helsinki. All data were obtained through the hospitals electronic medical records and outpatient follow-up system. The requirement for informed consent was waived due to the nature of retrospective study design.Detailed inclusion and exclusion criteria are presented in Additional file 1.

**Fig. 1:**
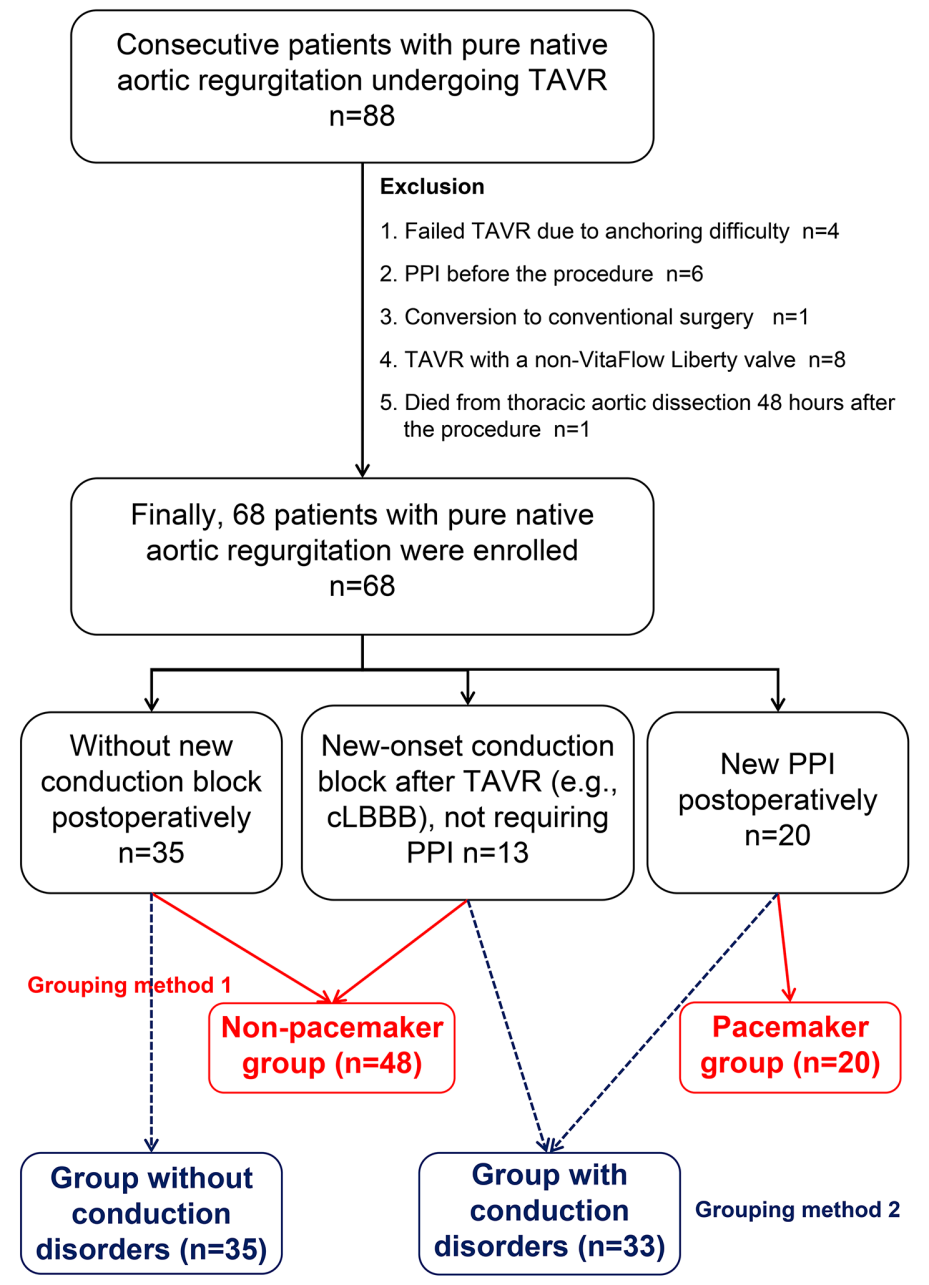
Flowchart of patient selection and grouping status PPI: permanent pacemaker implantation; cLBBB: complete left bundle branch block; TAVR: transcatheter aortic valve replacement

Based on the need for a pacemaker within 30 days after TAVR, patients were divided into the pacemaker and non-pacemaker groups (Fig. 1). Patients were also classified into the conduction block and non-conduction block groups, according to the presence or absence of new-onset LBBB or high-grade atrioventricular block (AVB) within 30 days after TAVR (Fig. 1).

### Data collection

**Fig. 2:**
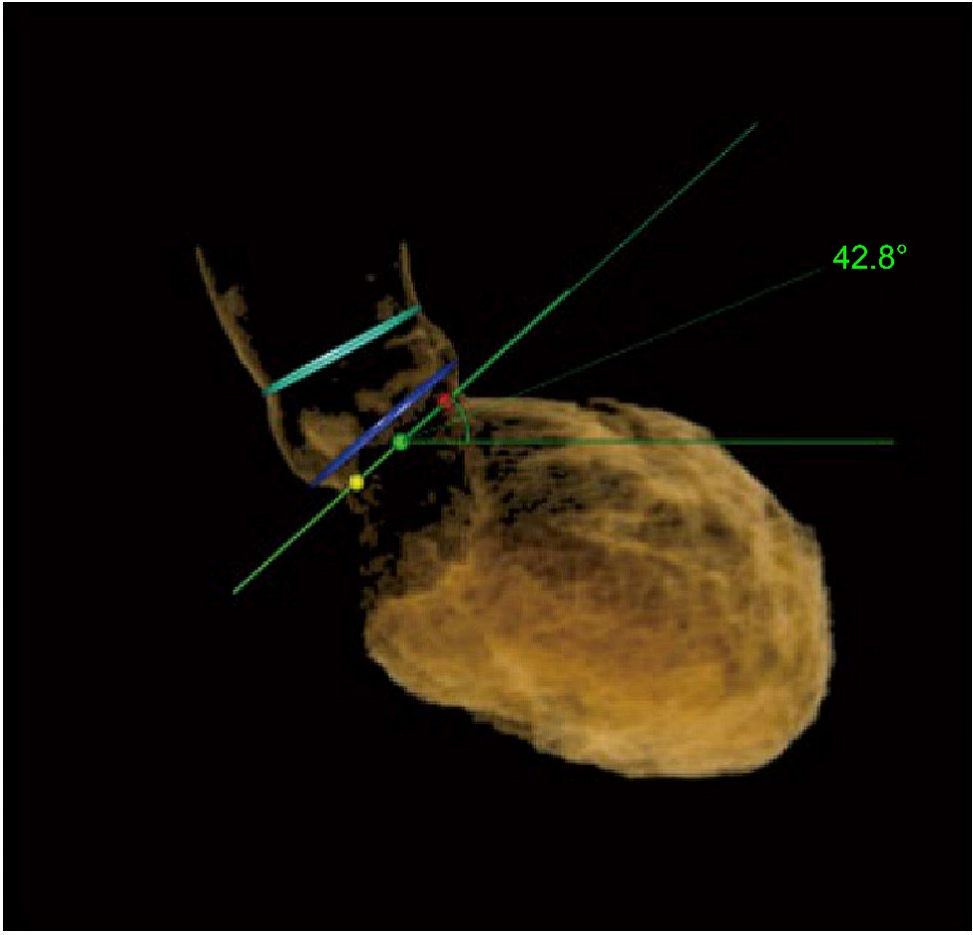
Illustration of the aortic root angle [26]

**Fig. 3:**
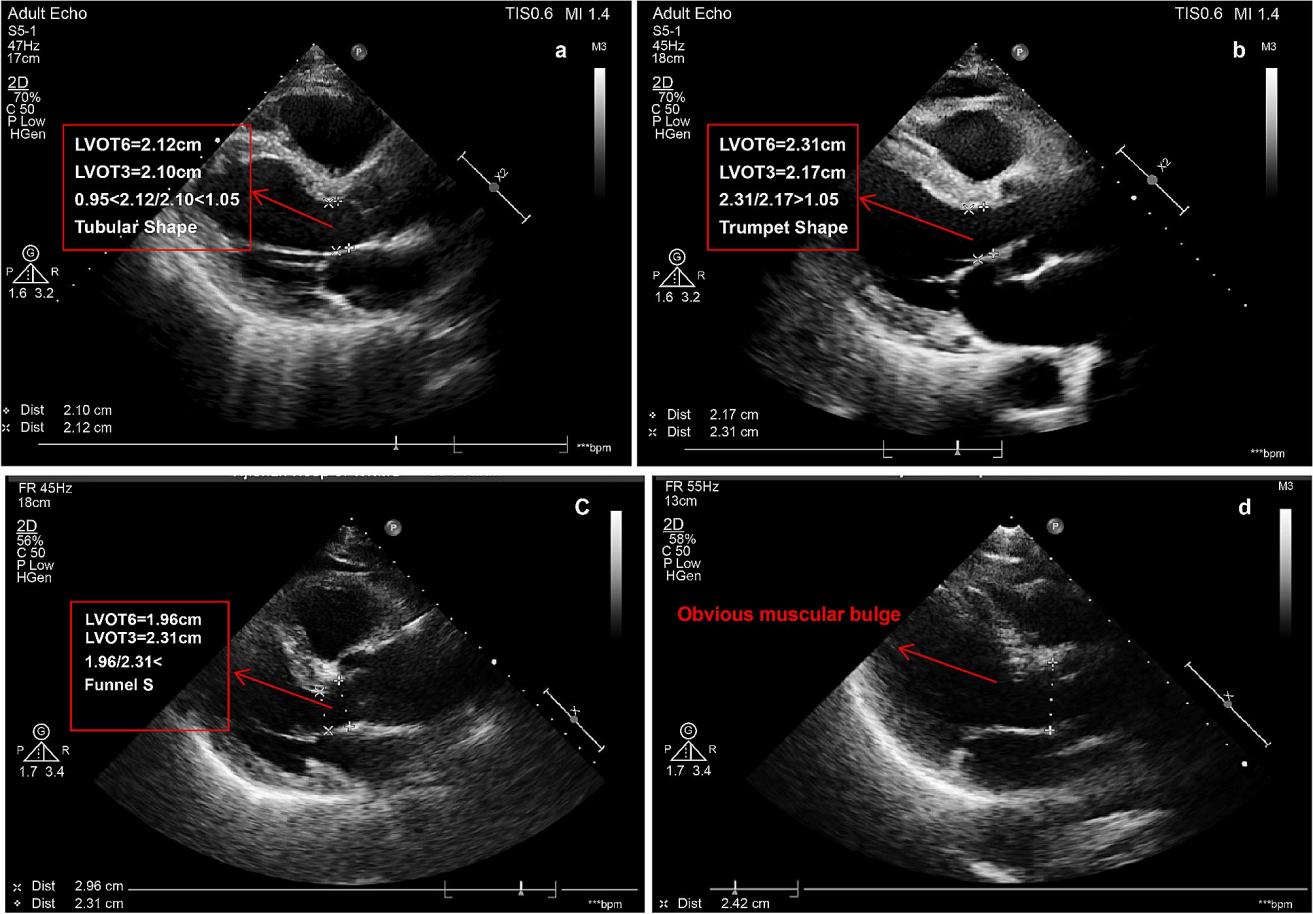
LVOT morphology detected by TTE **(a)** LVOT shape is tubular; **(b)** LVOT shape is like a trumpet; **(c)** LVOT shape is like a funnel; **(d)** LVOT shape is also like a funnel, as there is an obvious muscular bulge in the inner ventricular septum 6 mm below the aortic valve annulus. Here, the trumpet and funnel shapes were classified as non-tubular shapes; therefore, the LVOT in **b**, **c**, and **d** are all referred to as non-tubular shapes LVOT: ventricular outflow tract; TTE: transthoracic echocardiography

Preoperative computed tomography (CT) digital imaging data were analyzed using 3mensio Valves software (version 9.1, Pie Medical Imaging, Maastricht, Netherlands). We collected data on several parameters, such as mean diameter of the ascending aorta (AAO), sinotubular junction (STJ), aortic annulus (AA), left ventricular outflow tract (LVOT), and the angle of the aortic root (Fig. 2) [26]. LVOT morphology was assessed using preoperative CT and transthoracic echocardiography (TTE). To further clarify the morphology and measure the length of the mitral curtain, transesophageal echocardiography (TEE) was performed after anesthesia but before commencement of TAVR. According to the instruction manual for self-expanding valves used in the study, the valve should ideally be implanted at a depth of 0–6 mm below the AA. Therefore, we recorded the diameter and shape of the LVOT 6 mm below the AA and measured the LVOT diameter at 3 and 6 mm below the AA. A “tubular shape” morphology was defined as 0.95 ≤ LVOT6/LVOT3 ≤ 1.05, a “trumpet shape” morphology was defined as LVOT6/LVOT3 > 1.05, and a “funnel shape” morphology was defined as LVOT6/ LVOT3 < 0.95. In cases of an obvious muscular bulge in the inner ventricular septum 6 mm below the aortic valve annulus, we classified it as a “funnel shape” morphology (Fig. 3). For statistical analysis in this study, the trumpet and funnel shape morphologies were classified as “non-tubular shape” morphology.

After releasing the valve stent, we adjusted the imaging body position to ensure that the lowest point of the non-coronary sinus and the left and right coronary sinuses are at an equal level (cusp overlap section). Simultaneously, we made adjustments to align the bottom of the valve stent to the same equal level. Subsequently, aortic root angiography was performed, and the distance from the lower edge of the valve stent to the floor of the non-coronary cusp (NCC) was measured using DSA after control calibration.

**Fig. 4:**
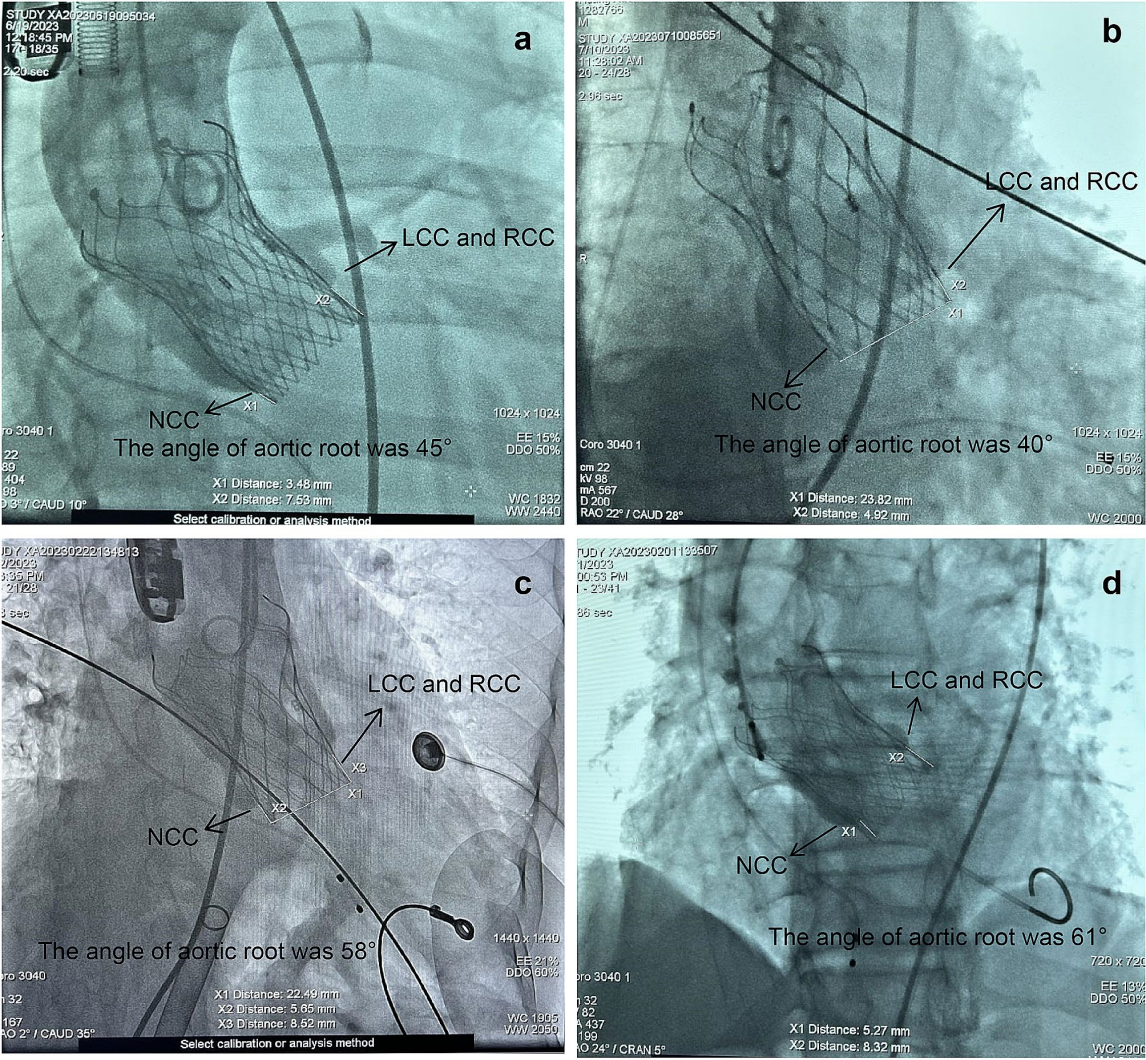
Valve implantation depth This depth is defined as the distance from the lower edge of the valve stent device after its release to the lowest point of the NCC, which is mostly consistent with the data measured by TEE **(a)** Valve implantation depth is 3.48 mm without new-onset heart block; **(b)** Valve implantation depth is 0.00 mm without new-onset heart block; **(c)** Valve implantation depth is 5.65 mm with new-onset heart block and need for PPI; **(d)** Valve implantation depth is 5.27 mm with new-onset heart block and need for PPI LCC: left coronary cusp; NCC: non-coronary cusp; RCC: right coronary cusp; TEE: transesophageal echocardiography

At present, there is no clear definition of valve implantation depth in TAVR-related guidelines [4, 5]. When performing TAVR in patients with PNAR, the lowest point of the NCC is used as the supporting and locating point for unfolding the valve stent device. Thus, we defined “valve implantation depth” as the distance from the lower edge of the valve stent device after its release to the lowest point of the NCC (Fig. 4). In this study, valve implantation depth was measured using TEE by a professional sonographer. In cases of discrepancy between the TEE and DSA measurement data, we used the DSA measurement data, considering the subjective nature of ultrasound. In this study, the TEE measurement data were consistent with the DSA measurement data in most patients.

Laboratory, echocardiographic, CT, preoperative and postoperative electrocardiography (ECG), and procedural and clinical data were collected immediately after TAVR and during hospitalization. These data included biological sex, age, body mass index (BMI), past medical history (e.g., history of hypertension and diabetes), high-sensitivity troponin level, creatine kinase-myoglobin level, brain natriuretic peptide (BNP) level, creatinine level, leukocyte count, red blood cell count, hemoglobin level, size of the left atrium, size of the LV, LVOT morphology, interventricular septal (IVS) thickness, left ventricle ejection fraction (LVEF), preoperative and postoperative ECG findings, and QRS wave width of ECG. In addition, the success rate of TAVR was recorded based on the Valve Academic Research Consortium-3 criteria [18, 27], new pacemaker implantation, postoperative AR including paravalvular leak and prosthetic valve regurgitation, and development of new complete LBBB (cLBBB).

### Study valve

**Fig. 5:**
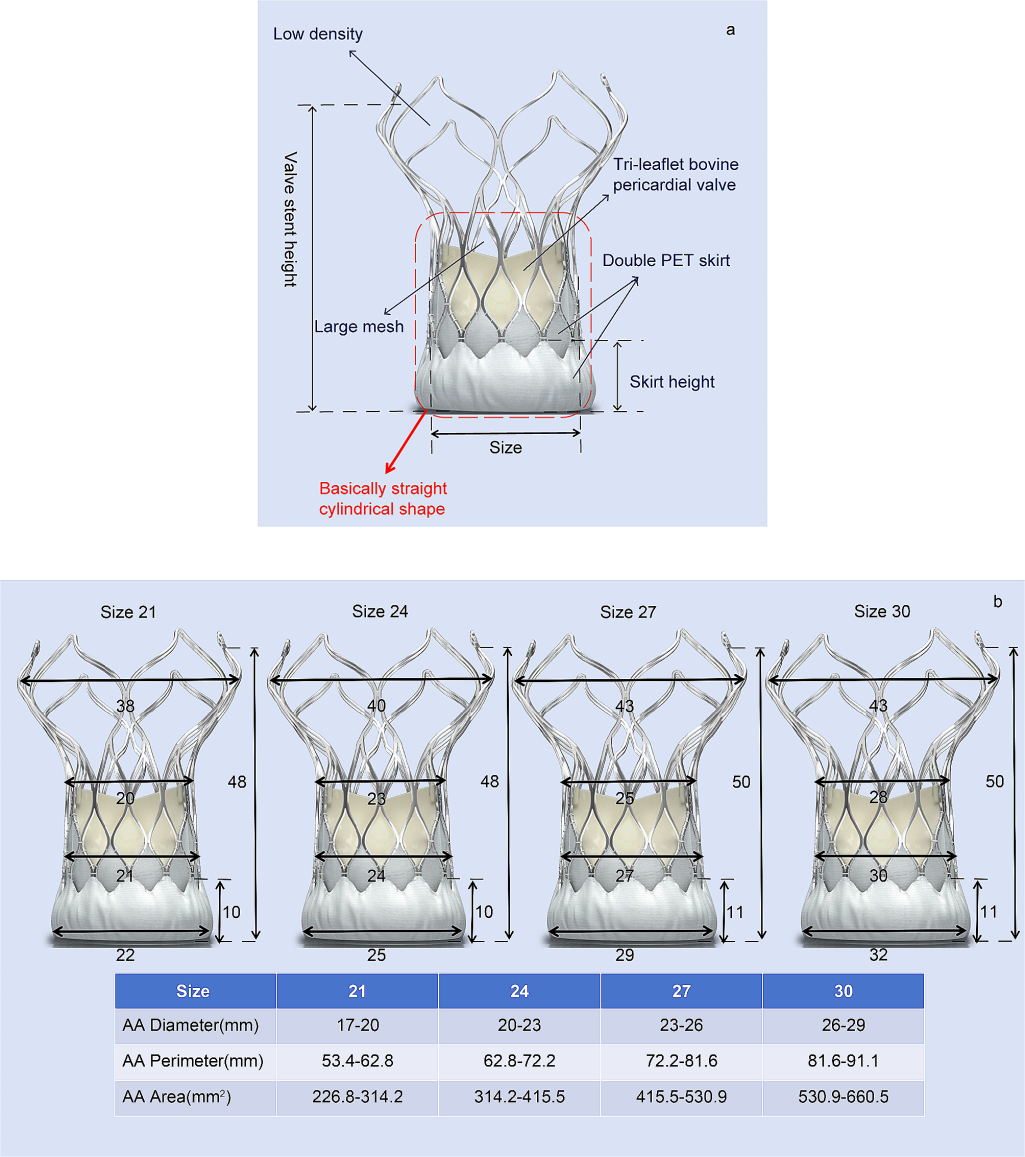
Characteristics of the new-generation VitaFlow™ valves **(a)** Schematic structure of the valve. **(b)** Available sizes of the VitaFlow™ valves The units of measurements in the above pictures are all in millimeters (mm). AA: aortic annulus; PET: polyethylene terephthalate

The valve used in this study is the new-generation VitaFlow™ valve manufactured by Shanghai MicroPort CardioFlow Medtech Co., Ltd. The valve device consists of a self-expanding nitinol frame and a tri-leaflet bovine pericardial valve. It incorporates an inner and outer double polyethylene terephthalate (PET) skirt at the LVOT and a mixed density bracket. Additionally, it utilizes bovine pericardium as valvular material, which has anticalcification properties. The double PET skirt is designed to reduce postprocedural paravalvular leak (PVL). The design incorporates a large mesh and low density at the ascending aorta section to facilitate easy crossing of the aortic arch and enable access to the coronary arteries (Fig. 5a) [28]. The VitaFlow™ valve has a straight cylindrical framework (Fig. 5a) [29]. The valve can be released using an electric handle, facilitating ease of use and allowing for smooth wire manipulation during deployment. Reinforced inner and outer shafts at the distal end of the delivery system are designed to provide stability and accurate deployment. The first-generation valve device (VitaFlow™) is not recapturable/repositionable [28], whereas the new-generation valve (VitaFlow Liberty™) delivery system adds a recyclable function through the motorized handle, combined with an exclusive double-bar spiral innovative structure, which not only ensures fast, stable, and accurate release and recovery of the valve but also offers flexibility and non-directionality in the delivery system. The valve prosthesis is manufactured in four different sizes (21, 24, 27, and 30 mm; Fig. 5b).

A multicenter study from China supported the efficacy and safety of the first-generation VitaFlow™ valve in treating patients with severe AS. The study reported low all-cause mortality, no major strokes, and no instances of moderate or severe PVL observed over a 12-month follow-up period [28]. Moreover, patients with bicuspid aortic valve had similar outcomes to those with tri-leaflet aortic valve [28]. In comparison to other self-expanding transcatheter heart valves, the first-generation VitaFlow™ valve is associated with a lower degree of AR, and fewer patients with AS undergoing TAVR with these valves develop moderate-to-severe AR [29]. Data from our hospital showed a lower new PPI rate (3.03%) after TAVR with the new-generation VitaFlow™ valve (VitaFlow Liberty™) in patients with AS [25].

### Statistical analysis

Basic descriptive measures were calculated for patient demographics and baseline laboratory, electrocardiographic, echocardiographic, CT measurement, and procedural data. The Shapiro–Wilk test was used to assess the normal distribution of quantitative data. Normally distributed quantitative data are reported as mean ± standard deviation, whereas other results are reported as median (P25, P75). Continuous data were initially compared between different groups using the Mann–Whitney U-test or independent samples *t*-test, whereas categorical variables were compared using Fisher’s exact test or the chi-square test. Next, the variables were examined in univariate regression models to identify predictors of postoperative PPI and cLBBB or high-grade AVB in patients. Subsequently, a multivariate logistic regression analysis was performed incorporating the significant variables from the univariate analyses. Considering sample size matching, we strictly included variables with *P* < 0.05 into the multivariate logistic regression analysis model. Receiver operating characteristic (ROC) curve was used to evaluate the implantation depth of the valve stent device and angle of the aortic root as predictors of cLBBB or high-grade AVB. The appropriate index cutoff value for valve implantation depth and angle of the aortic root were selected using Youden’s index. A *P*-value of < 0.05 was considered statistically significant.

## Results

A total of 88 patients with PNAR were treated with TAVR at our hospital from November 2021 to June 2023. Twenty patients were excluded from the study as follows: four patients with valve implantation failure due to an inability to anchor it intraoperatively, six had a preoperatively fitted pacemaker, one required transfer to surgery because the valve slipped into the LV while releasing it intraoperatively, eight had a non-VitaFlow Liberty valve, and one patient died in the hospital 48 h after the procedure due to a postoperative complication of thoracic aortic dissection. The remaining 68 patients met the inclusion criteria. Figure 1 presents the patient flow chart.

Out of 88 patients with PNAR, TAVR was unsuccessful in five cases, resulting in a success rate of 94.31%. Among the 83 patients who underwent a successful procedure, moderate or above-moderate AR was reported in four of them after the procedure, accounting for an incidence of 4.82%.

Twenty patients were newly implanted with a permanent pacemaker after TAVR, accounting for an incidence of 29.41%. Based on univariate analyses of baseline demographics, laboratory, electrocardiographic, echocardiographic, and CT measurement, and procedure data, and preoperative BNP levels, significant differences were noted in the presence or absence of preoperative complete right bundle branch block (cRBBB) or first-degree AVB and LVOT morphology between the pacemaker and non-pacemaker groups (Table 1). With inclusion of the three aforementioned significantly different indicators in the multivariate logistic regression analysis, presence of preoperative cRBBB or first-degree AVB [odds ratio (OR) = 31.44, 95% confidence interval (CI): 1.218–811.513, *P* = 0.038] and non-tubular LVOT (OR = 12.05, 95% CI: 1.124–129.199, *P* = 0.04) remained independent risk factors for pacemaker implantation (Table 2). Patients’ demographics and baseline data were also analyzed and compared between the two conduction disorder groups. Only LVOT type, aortic root valve angle, and valve implantation depth were significantly different between the two groups (*P* < 0.05; Table 3). After multiple logistic regression analysis that included the three aforementioned significantly different indicators, valve implantation depth (OR = 1.671, 95% CI: 1.189–2.348, *P* = 0.003) and angle of the aortic root (OR = 1.116, 95% CI: 1.012–1.230, *P* = 0.028) remained independent risk factors for cLBBB or high-grade AVB (Table 4).

**Table 1:** Baseline data, test results, echocardiographic data, and computed tomography measurements of the two patient groups

Variables	Non-pacemaker group (*n* = 48)	Pacemaker group (*n* = 20)	***P*** value
Age (years)	74.59 ± 7.805	74.20 ± 9.18	0.902
Sex (male)	28 (58.3%)	7 (35%)	0.111
BMI (kg/m^2^)	21.03 ± 2.57	21.13 ± 3.048	0.925
STS score	7.68 ± 1.25	8.40 ± 1.96	0.306
**Past medical history**			
Hypertension	34 (70.8%)	11 (55.0%)	0.264
Coronary heart disease	10 (20.8%)	7 (35%)	0.235
Diabetes	4(8.3%)	6 (30%)	0.054
**Laboratory data**			
Leukocyte count (10^9^/L)	5.24 ± 1.63	5.18 ± 0.88	0.919
Red blood cell count (10^12^/L)	3.93 ± 0.50	3.84 ± 0.56	0.636
Hemoglobin level (g/L)	117.91 ± 15.83	116.10 ± 16.93	0.771
Platelet count (10^9^/L)	158.09 ± 58.55	156.60 ± 51.58	0.945
Total cholesterol level (mmol/L)	3.75 ± 0.95	3.74 ± 1.33	0.985
Low-density cholesterol level (mmol/L)	2.03 ± 0.81	2.10 ± 0.84	0.843
Creatinine level (µmol/L)	83.64 ± 30.27	95.76 ± 47.18	0.387
Fasting glucose level (mmol/L)	4.68 ± 0.429	5.18 ± 0.72	0.064
High-sensitivity troponin level (ng/mL)	0.011 (0.009,0.025)	0.009 (0.004,0.183)	0.131
CKMB level (U/L)	15.82 ± 6.80	11.60 ± 5.66	0.098
BNP level (pg/mL)	329.00 (144.00,848.75)	89.50 (63.00,251.00)	0.040
Electrocardiography findings			
Preoperative QRS width (ms)	102.05 ± 20.14	114.20 ± 25.36	0.155
Preoperative cRBBB or first-degree AV block	2 (4.2%)	10 (50%)	< 0.001
**Echocardiography**			
LA (mm)	44.32 ± 4.99	40.30 ± 8.53	0.103
LV (mm)	56.55 ± 8.37	56.90 ± 8.77	0.914
IVS (mm)	9.89 ± 1.88	9.9 ± 1.91	0.985
LVEF (%)	52.95 ± 9.69	52.90 ± 9.53	0.988
Mitral curtain length (mm)	4.86 ± 1.25	5.00 ± 0.94	0.761
LVOT type (non-tubular)	14 (29.2%)	16 (80.0%)	< 0.001
**CT data**			
Mean diameter of AA (mm)	24.66 ± 2.23	24.16 ± 3.14	0.607
Mean diameter of LVOT (mm)	24.96 ± 3.19	24.20 ± 4.01	0.566
Mean diameter of STJ (mm)	35.32 ± 3.12	33.99 ± 4.45	0.336
Angle of the aortic root (°)	55.50 ± 10.25	59.90 ± 8.25	0.243
Mean diameter of AAO (mm)	43.17 ± 3.04	40.23 ± 5.56	0.061
AA calcification	6 (12.5%)	3 (15.0%)	1
LVOT calcification	2 (4.2%)	2 (10%)	0.575
**Valve device and TAVR intervention procedures date**			
Valve device type (mm)	28.66 ± 1.78	28.32 ± 2.28	0.586
Valve implantation depth (mm)	2.44 (0.00, 5.06)	4.73 (2.46, 6.29)	0.118
**Other**			
Largest diameter of the upper corolla of the valve device / diameter of AAO	0.99 ± 0.067	1.08 ± 0.14	0.090
Diameter of the inferior edge of valve device/diameter of aortic annular	1.24 ± 0.07	1.25 ± 0.09	0.672
Diameter of the inferior edge of valve device/Mean diameter of LVOT	1.23 ± 0.11	1.26 ± 0.14	0.553
Mean diameter of LVOT/Mean diameter of aortic annular	1.01 ± 0.07	1.00 ± 0.06	0.643

BMI: body mass index; BNP: brain natriuretic peptide; cRBBB: complete right bundle branch block; CKMB: creatine kinase-myoglobin binding; LVOT: left ventricular outflow tract; CT: computed tomography; STJ: sinotubular junction; LA: left atrium; LV: left ventricle; IVS: interventricular septal; LVEF: left ventricular ejection fraction; AAO: ascending aorta

**Table 2:** Multifactor analysis results of pacemaker placement after transcatheter aortic valve replacement

Variable	OR	95% CI	***P*** value
LVOT type (non-tubular)	12.05	1.124–129.199	0.04
Preoperative cRBBB or first-degree AV block	31.442	1.218–811.513	0.038

LVOT type, Preoperative cRBBB or first-degree AV block, and BNP were included in the multivariate logistic regression analysis

OR: odds ratio; CI: confidence interval; cRBBB: complete right bundle branch block; AV: atrioventricular

**Table 3:** Baseline data, test results, electrocardiography findings, echocardiographic data, and computed tomography measurements in patient groups

Variable	Group without conduction disorders (*n* = 35)	Group with conduction disorders (*n* = 33)	***P*** value
Sex (male)	21 (60.0%)	14 (42.4%)	0.225
Age (years)	76.63 ± 7.42	72.31 ± 8.4.2	0.135
BMI (kg/m^2^)	21.25 ± 2.70	20.87 ± 2.72	0.697
STS score	7.69 ± 1.25	8.13 ± 1.75	0.422
Past medical history			
Hypertension	24 (68.6%)	21 (63.6%)	0.799
Coronary heart disease	7 (20.0%)	10 (30.3%)	0.406
Diabetes	3 (8.6%)	7 (21.2%)	0.259
Laboratory data			
Leukocyte count (*10^9^/L)	5.61 ± 1.62	4.83 ± 1.11	0.119
Red blood cells count(*10^12^/L)	3.94 ± 0.58	3.87 ± 0.46	0.694
Hemoglobin level (g/L)	117.13 ± 18.3	117.56 ± 13.75	0.940
Platelet count (*10^9^/L)	157.50 ± 60.54	157.75 ± 52.25	0.990
Total cholesterol level (mmol/L)	3.73 ± 0.82	3.77 ± 1.29	0.913
Low-density cholesterol level (mmol/L)	2.00 ± 0.72	2.10 ± 0.91	0.736
Creatinine level (µmol/L)	89.08 ± 30.70	85.78 ± 41.68	0.800
Fasting glucose level (mmol/L)	4.66 ± 0.55	4.96 ± 0.78	0.224
High-sensitivity troponin level (ng/mL)	0.009 (0.008,0.020)	0.010 (0.005,0.028)	0.742
CKMB level (U/L)	16.05 ± 4.71	13.86 ± 3.59	0.095
BNP level (pg/mL)	294 (100,766)	260 (156,425)	0.368
Electrocardiography findings			
Preoperative QRS width (ms)	104.06 ± 21.82	107.63 ± 23.2	0.658
Preoperative cRBBB or first-degree AV block	4 (11.4%)	8 (24.2%)	0.166
Echocardiography			
LA (mm)	44.56 ± 5.27	41.56 ± 7.30	0.193
LV (mm)	55.05 ± 7.29	59.57 ± 9.51	0.086
IVS (mm)	9.97 ± 1.32	9.81 ± 2.32	0.817
LVEF (%)	52.25 ± 8.70	53.63 ± 10.46	0.689
Mitral curtain length	4.88 ± 1.20	4.94 ± 1.12	1
LVOT type (non-tubular)	14 (29.6%)	16(80%)	0.008
CT data			
Mean diameter of AA (mm)	24.29 ± 2.19	24.72 ± 2.84	0.639
Mean diameter of LVOT (mm)	24.80 ± 3.21	2465 ± 3.72	0.904
Mean diameter of STJ (mm)	35.13 ± 3.39	34.68 ± 3.84	0.728
Angle of the aortic root (°)	55.23 ± 5.99	61.48 ± 8.72	0.009
Mean diameter of AAO (mm)	42.96 ± 2.67	41.55 ± 5.22	0.345
AA calcification	4 (11.4%)	5 (15.2%)	0.730
LVOT calcification	2 (5.7%)	2 (6.1%)	1
Valve device and TAVR intervention procedure dates			
Valve device type (mm)	28.8 ± 1.897	28.6 ± 1.92	0.776
Valve implantation depth (mm)	0.00 (0.00, 4.00)	5.92 (3.31 6.13)	0.001
Others			
Largest diameter of the upper corolla of the valve device/diameter of AAO	1.00 ± 0.06	1.05 ± 0.12	0.170
Diameter of the inferior edge of valve device/diameter of aortic annular	1.25 ± 0.07	1.23 ± 0.08	0.618
Diameter of the inferior edge of valve device/Mean diameter of LVOT	1.24 ± 0.11	1.25 ± 0.13	0.705
Mean diameter of LVOT/Mean diameter of aortic annular	1.02 ± 0.07	1.00 ± 0.06	0.308

BMI: body mass index; BNP: brain natriuretic peptide; cRBBB: complete right bundle branch block; CKMB: creatine kinase-myoglobin binding; LVOT: left ventricular outflow tract; CT: computed tomography; STJ: sinotubular junction; LA: left atrium; LV: left ventricle: IVS: interventricular septal; LVEF: left ventricular ejection fraction

**Table 4:** Multifactor analysis results for predicting left bundle branch block or high atrioventricular block

Variable	OR	95% CI	***P*** value
Valve implantation depth (mm)	1.671	1.189–2.348	0.003
Angle of the aortic root (°)	1.116	1.012–1.230	0.028

Valve implantation depth, Angle of the aortic root (°), and LVOT type were included in the multivariate logistic regression analysis

OR: odds ratio; CI: confidence interval

**Fig. 6:**
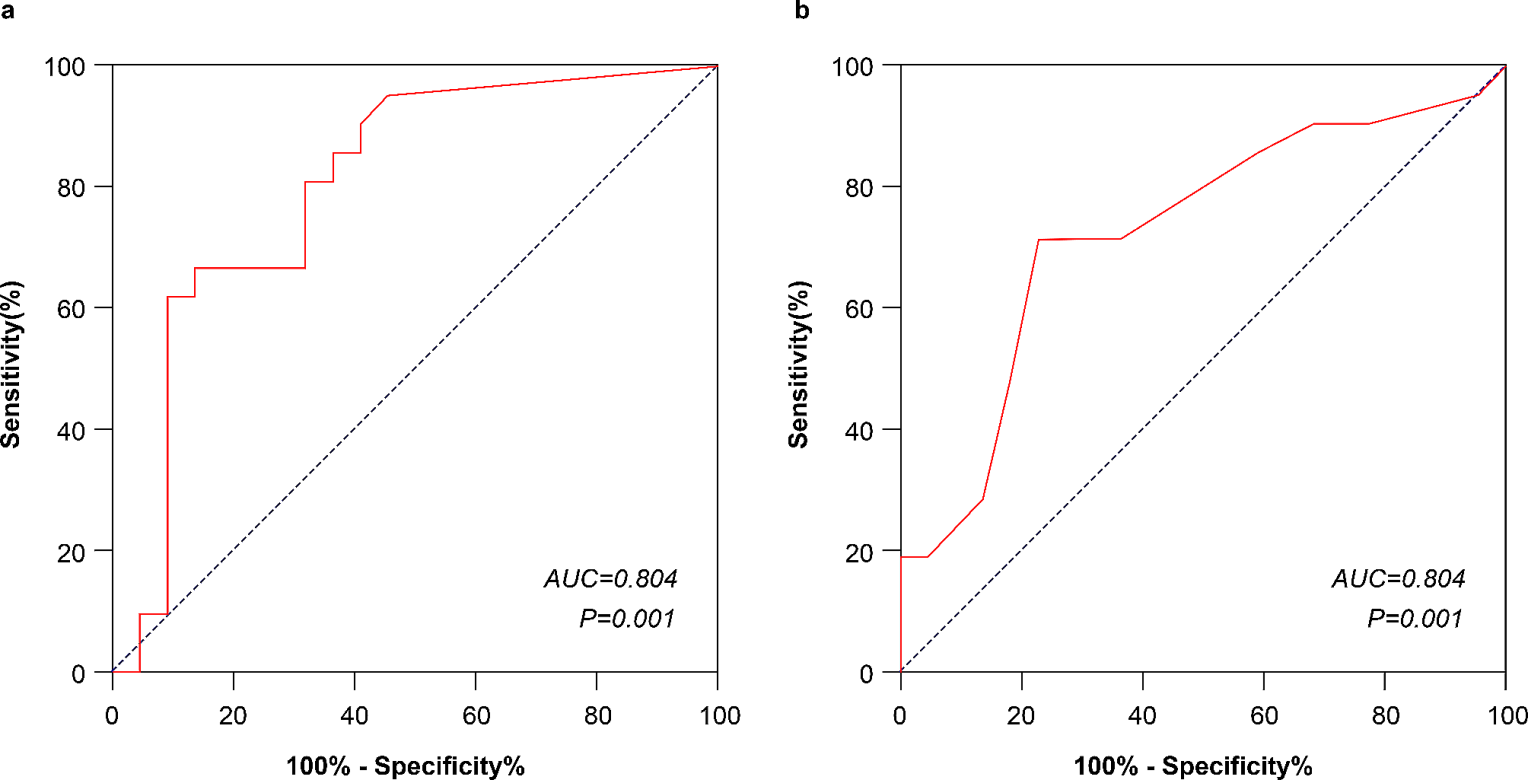
ROC curves **(a)** ROC curve for valve implantation depth for predicting postoperative LBBB or high-grade AVB, with AUC = 0.804, 95% CI: 0.666–0.942, *P* = 0.001, cutoff point according to Youden’s index of 4.30 mm, sensitivity of 0.667, and specificity of 0.864; **(b)** ROC curve for the angle of the aortic root for predicting postoperative LBBB or high-grade AVB, with AUC = 0.726, 95% CI: 0.570–0.882, *P* = 0.011, cutoff point according to Youden’s index of 59°, sensitivity of 0.714, and specificity of 0.773 AUC: area under the curve; AVB: atrioventricular block; LBBB: left bundle branch block; ROC: receiver operating characteristic

Figure 6a presents the ROC curve for valve implantation depth as a predictor of cLBBB or high-grade AVB, using a cutoff value of 4.30 mm for valve implantation depth with a sensitivity of 61.9% and specificity of 90.9%. The area under the curve (AUC) of the ROC for valve implantation depth in predicting the new-onset of cLBBB or high-grade. AVB was 0.804 (95% CI: 0.666–0.942, *P* = 0.001). Further, Fig. 6b displays the ROC curve for angle of the aortic root as a predictor of cLBBB or high-grade AVB, using a cutoff value of 59° for the angle of the aortic root with a sensitivity of 71.4% and a specificity of 77.3%. The AUC of the ROC for angle of the aortic root in predicting the new-onset of cLBBB or high-grade AVB was 0.826, with a 95% CI of 0.570–0.882 and *P* = 0.011.

## Discussion

As the population ages, there is an increasing incidence of aortic valve disease. An epidemiological survey revealed that the prevalence of moderate-to-severe PNAR is approximately 1.2% in China, making it the most common type of valvular heart disease in the nation [30]. With the utilization of a new generation of self-expanding valves, combined with the growing experience of TAVR procedures and technological advancements, the success rate of TAVR in treating patients with PNAR has significantly increased. Moreover, many complications, such as PVR and moderate-to-severe AR, have been significantly reduced [13]. However, the occurrence of new PPI or new-onset conduction disorders, such as LBBB, has not been reduced in patients with PNAR after TAVR [13, 14, 16–19], which seriously affects the prognosis of patients [14, 23, 24].

TAVR-related conduction disturbances, mainly new-onset LBBB and advanced AVB requiring PPI, remain the most common complication of this procedure [31]. Therefore, this study aimed to identify predictors of new-onset conduction block post-TAVR using new-generation self-expanding valves (VitaFlow Liberty™) in patients with PNAR. Our study revealed a 94.31% success rate of TAVR in patients with PNAR, with a 4.82% incidence of above-moderate AR after the procedure. Among patients with PNAR undergoing TAVR using self-expanding valves, preoperative cRBBB or first-degree AVB and a non-tubular LVOT were identified as indicators associated with a higher likelihood of requiring PPI. Additionally, deeper valve implantation depth and a greater angle of aortic root may serve as independent risk factors for new-onset cLBBB or high-grade AVB post-TAVR.

Chen et al. used first-generation self-expanding valves manufactured in China to perform TAVR in patients with PNAR and reported that VitaFlow™, which is a self-expanding valve with straight cylindrical shape (Fig. 5a), improved the success rate of TAVR compared with another self-expanding valve without a straight cylindrical shape [26]. Similarly, our results suggest that the use of VitaFlow Liberty™ can further improve the success rate of TAVR in patients with PNAR. The high success rate in our study might be attributed to the special aortic root anatomy of patients with PNAR and the self-expanding valve we used with a straight cylindrical shape (Fig. 5a). Typically, these patients have a large valve annulus without annular or valve leaflet calcification, which often necessitates anchoring the valve device in conjunction with LVOT [32]. The lower section of the VitaFlow™ valve device is essentially a straight cylindrical shape (Fig. 5a), offering increased contact area and friction between the valve device and the LVOT, thereby increasing the radial support force and facilitating valve anchoring [26]. Moreover, the new-generation VitaFlow™ valve system used in this study has recovery and repositioning functions, which also helps improve the success rate of TAVR in PNAR.

We found that preoperative cRBBB or first-degree AVB is an independent risk factor for PPI after TAVR in patients with PNAR, which is consistent with the results of a previous study on PPI after TAVR for AS [33]. However, to the best of our knowledge, this study is the first to show that non-tubular LVOT is also an independent risk factor for PPI in patients with PNAR after TAVR. We found that the non-tubular morphology of LVOT is mainly characterized by a flared or funnel shape. With a flared LVOT, the valve stent is prone to penetrate too deeply into the LVOT tract, leading to a conduction block. In contrast, with a funnel-shaped LVOT, the compression ratio of the lower valve stent edge is too large after successful implantation, and the radial force squeezing the outflow tract is too strong, leading to dysfunction of the bundle of His or LBB. Further randomized controlled studies are required to verify these results.

**Fig. 7:**
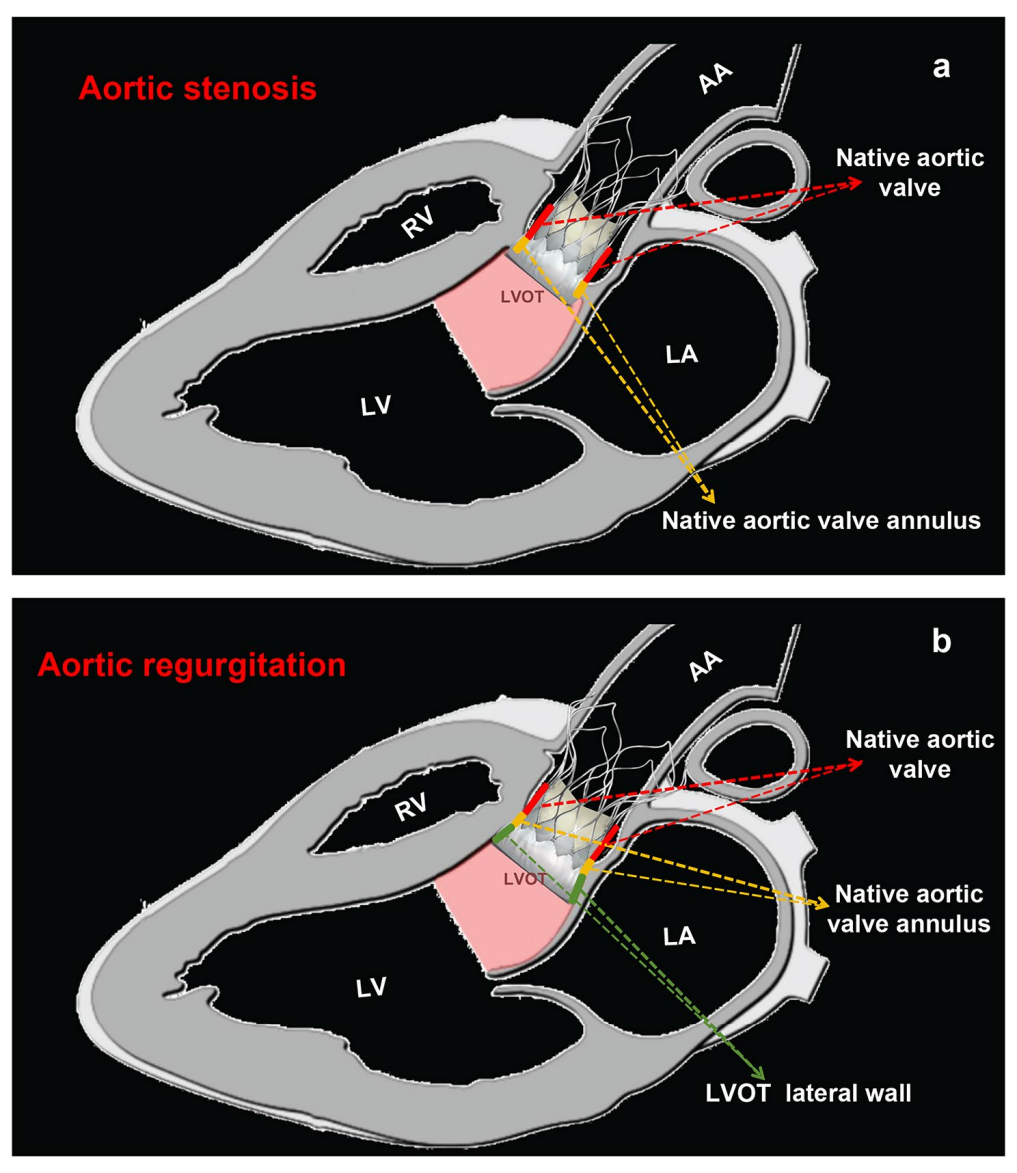
Different anchoring mechanisms of self-expanding valves during TAVR in patients with AS and PNAR **(a)** Anchoring of TAV prosthesis in patients with AS mainly relies on aortic annulus, calcified valve leaflets, and STJ; **(b)** anchoring of TAV prosthesis in PNAR relies on LVOT, aortic annulus, thickened aortic valve leaflet (if there is a thickening of the valve leaflets), and STJ. AA: ascending aorta; AS: aortic stenosis; LA: left atrium; LV: left ventricle; LVOT: left ventricular outflow tract; PNAR: pure native aortic regurgitation; RV: right ventricle; STJ: sinotubular junction; TAV: transcatheter aortic valve; TAVR: TAV replacement

In addition, treatment of PNAR with TAVR poses unique technical challenges. Unlike patients with AS, most patients with PNAR do not have calcified valves and have a significantly dilated annulus and left ventricle, making fixation of the transcatheter valve prosthesis in the plane of the valve extremely difficult [34]. Thus, anchoring the transcatheter aortic valve prosthesis in patients with AS mainly relies on the aortic annulus, calcified valve leaflets, and STJ (Fig. 7a). In contrast to AS, the anchoring of transcatheter aortic valve prosthesis in PNAR relies on LVOT, aortic annulus, thickened aortic valve leaflet (if present), and STJ (Fig. 7b) [26, 32]. This may explain why, in our study, the risk factors for new PPI after TAVR differ between patients with PNAR and those with AS.

In line with previous studies, our study found a higher rate of new-onset conduction block in patients with PNAR after TAVR [13, 26]. The presence of new-onset conduction block after the procedure has significant effects on patient prognosis [14, 24]. In our study, greater aortic root angle and deeper valve implantation depth were independent risk factors for cLBBB or high-grade AVB. The importance of valve implantation depth was further supported by our ROC analysis findings, which are consistent with previous results on new-onset conduction block after TAVR for AS [33]. We also found a significantly increased risk of cLBBB or high-grade AVB with increased aortic root angle. The ROC analysis also further supported the predictive ability of aortic root angle for new conduction block after TAVR in patients with PNAR. The Youden index indicated that the sensitivity and specificity of predicting new conduction block post-TAVR were 0.714 and 0.773, respectively, when the angle of the aortic root was > 59°.

When performing TAVR in patients with PNAR using self-expanding valves, owing to the longer stent frame, the lowest point of the NCC serves as the initial supporting point for unfolding the valve stent device, and the valve is subsequently unfolded outward [35]. Finally, the prosthesis comes in contact with the side of the left coronary cusp (LCC), completing the valve release. Therefore, deployment of self-expanding valves is usually asymmetric [36]. Moreover, in our study, a smaller aortic root angle was associated with improved coaxiality and better alignment between the valve stent device and the aortic root, i.e., with a smaller angle between the plane of the lower valve stent device edge and the annular plane. In contrast, a larger aortic root angle was associated with worse coaxiality and poor alignment between the valve stent device and the aortic root, i.e., with a larger angle between the plane of the lower valve stent device edge and the annular plane and corresponding to an increased depth of the valve stent at the lower edge of the LCC and right coronary cusp (RCC). The close proximity between the heart conduction system and the aortic valve complex can explain the genesis of perioperative conduction disturbances during TAVR [37–39]. Conduction disorders primarily result from direct mechanical damage to the conduction system, associated with ischemia, hematoma, and varying degrees of edema, occurring during the deployment of TAVR [40]. The triangle of Koch, defined by the ostium of the coronary sinus, the insertion point of the tricuspid valve septal leaflet, and the tendon of Todaro, is often used as an anatomical marker for the position of the atrioventricular node (AVN) within the right atrium [39, 41–43]. The AVN continues as the bundle of His, penetrating to the left through the central fibrous body and piercing the membranous septum (MS). The position of the bundle of His is typically adjacent to the infra-anterior border of the MS, covered by a roof of ventricular muscle and ascending obliquely from the infra-posterior to supra-anterior directions [37, 39]. The MS is in conjunction with the RCC and NCC of the aortic valve [37]. There is considerable individual variability in MS length and its anatomical relationship with the bundle of His and the AVN [37]. Typically, the bundle of His penetrates the distal borders of the MS, adjacent to the transition to the muscular part of the IVS, and then separates into a left and right bundle branch underneath the surface of the LVOT [31, 39]. Kawashima and Sato showed that the area at greatest risk of injury during TAVR is the side of the MS adjacent to the RCC [37]. In summary, we can conclude that the His and LBB bundles are typically located in the LVOT below the RCC or at the LVOT below the junction of NCC and RCC. This positioning makes them susceptible to being compressed, potentially leading to a new-onset conduction block in the setting of TAVR. Thus, a greater aortic root angle corresponds to a deeper position of the valve stent at the lower edge of the LCC and RCC, which is consistent with the effect of valve implantation depth on the conduction system after TAVR. Based on the valve implantation depth, as defined in our study, an increased depth corresponded to a deeper position of the valve stent at the lower edge of the LCC and RCC. However, even at a normal valve implantation depth, a larger aortic root angle often results in a deeper device depth below the LCC and RCC (Fig. 4c and d). This may explain why a larger aortic root angle was identified an independent risk factor for cLBBB or high-grade AVB after TAVR in patients with PNAR in this study, which requires further validation in subsequent studies. However, to the best of our knowledge, this study is the first to show that the angle of the aortic root is also an independent risk factor for new-onset heart block in patients with PNAR who have undergone TAVR with self-expanding valves. Furthermore, this finding provides valuable insights into the need for optimizing the intervention process and the equipment used to enhance the coaxial alignment between the valve stent and the aortic root. Thus, we can effectively reduce the occurrence of new-onset heart block post-TAVR in patients with PNAR.

In summary, our study found that preoperative cRBBB, first-degree AVB, and non-tubular LVOT were closely associated with the need for PPI after TAVR in patients with PNAR. Moreover, the valve implantation depth and aortic root angle were closely associated with new-onset LBBB or high-grade AVB in these patients. Therefore, before performing TAVR in patients with PNAR, the possibility of new PPI or postoperative new-onset conduction block should be evaluated by integrating ECG, LVOT morphology, and aortic root angle measurements. Thus, based on the specific patient conditions, more optimal transcatheter interventional therapy strategies, such as valve implantation depth and valve device selection, may be planned.

### Limitations

This study has several limitations. First, this was a single-center retrospective study with a limited sample size and was performed using only a single type of self-expanding valve; therefore, our findings require further validation in a larger multicenter cohort using various types of self-expanding valves. Second, the intraoperative valve stent implantation depth was measured using DSA software calibration combined with intraoperative TEE rather than with postoperative CT; therefore, some errors may exist. Finally, LVOT morphology was assessed manually and subjectively using preoperative TTE, intraoperative TEE, and preoperative CT data rather than digital modeling through CT or magnetic resonance imaging.

## Conclusions

When using a self-expanding valve for TAVR in patients with PNAR, preoperative cRBBB or first-degree AVB on ECG and non-tubular LVOT measured by TTE were associated with the need for new PPI. Further, intraoperative valve implantation depth and aortic root angle were also associated with postoperative new-onset cLBBB or high-grade AVB. Therefore, in cases wherein the preoperative ECG shows cRBBB or first-degree AVB, the LVOT is non-tubular, and the aortic root angle is large (i.e., horizontal heart), we should comprehensively assess whether to perform TAVR or optimize the procedure based on the patient’s condition, such as by minimizing the valve implantation depth under the NCC prior to valve device release, to avoid the occurrence of postoperative new-onset conduction block.

### Electronic supplementary material

Below is the link to the electronic supplementary material.

Supplementary Material 1

## Data Availability

The original contributions presented in the study are included in the article/supplementary material; further inquiries can be directed to the corresponding author/s.

## Abbreviations

AA: aortic annulus
AAO: ascending aorta
AR: aortic regurgitation
AS: aortic stenosis
AVB: atrioventricular block
AVN: atrioventricular node
AUC: area under the curve
BMI: body mass index
BNP: brain natriuretic peptide
CI: confidence interval
cLBBB: complete left bundle branch block
cRBBB: complete right bundle branch block
CT: computed tomography
DSA: digital subtraction angiography
ECG: electrocardiography
IVS: interventricular septal
LBBB: left bundle branch block
LCC: left coronary cusp
LV: left ventricle
LVEF: left ventricle ejection fraction
LVOT: left ventricular outflow tract
MS: membranous septum
NCC: non-coronary cusp
OR: odds ratio
PET: polyethylene terephthalate
PNAR: pure native aortic regurgitation
PPI: permanent pacemaker implantation
PVL: paravalvular leak
RCC: right coronary cusp
ROC: receiver operating characteristic
STJ: sinotubular junction
TAVR: transcatheter aortic valve replacement
TTE: transthoracic echocardiography
