# Supplementary material for: Predictors for new-onset conduction block in patients with pure native aortic regurgitation after transcatheter aortic valve replacement with a new-generation self-expanding valve (VitaFlow Liberty™): a retrospective cohort study
Source: BMC Cardiovasc Disord. 2024 Jan 28;24:77. doi: 10.1186/s12872-024-03735-z (PMC10822180; doi:10.1186/s12872-024-03735-z)
Supplement: Supplementary file 1 — Supplementary Material 1 [file 12872_2024_3735_MOESM1_ESM.docx]

**Additional file 1**

**Predictors for New-Onset Conduction Block in Patients with Pure Native Aortic Regurgitation After Transcatheter Aortic Valve Replacement with a New-Generation Self-Expanding Valve (VitaFlow LibertyTM): A Retrospective Cohort Study**

Xuan Zhang ^a†^, Cheng Liang ^a†^, Lintao Zha ^a*^, Quan Zuo ^a^, Guobing Hu ^b^, Jie Ding ^b^, Shengxing Tang ^a*^

^a^ Department of Cardiology, The First Affiliated Hospital (Yijishan Hospital) of Wannan Medical College, Wuhu, Anhui, China

^b^ Department of Ultrasound Medicine, The First Affiliated Hospital (Yijishan Hospital) of Wannan Medical College, Wuhu, Anhui, China

^†^These authors share first authorship.

*** Corresponding authors:**Lintao Zha

Department of ~~Cardiovascular Medicine~~ Cardiology

The First Affiliated Hospital (Yijishan Hospital) of Wannan Medical College

No. 2, Zheshan West Road, Jinghu District, Wuhu City, Anhui Province, China

Tel: 13721211787

Fax: 0553-5739551

Email: [qdyzlt@163.com](mailto:qdyzlt@163.com)

Shengxing Tang

Department of ~~Cardiovascular Medicine~~ Cardiology

The First Affiliated Hospital (Yijishan Hospital) of Wannan Medical College

No. 2, Zheshan West Road, Jinghu District, Wuhu City, Anhui Province, China

Tel: 13955330717

Fax: 0553-5739551

Email: [yjsyytsx@163.com](mailto:yjsyytsx@163.com)

**Supplementary Methods**

**Inclusion and Exclusion Criteria**

The following inclusion criteria were applied: (1) patients diagnosed with severe PNAR on transthoracic echocardiography (TTE), according to the guidelines for valve disease management [1-3]; (2) patients who underwent computed tomography (CT) before TAVR for analysis of their conditions, such as the aortic valve root and size of the left ventricular outflow tract (LVOT); (3) patients who underwent transesophageal echocardiography (TEE) after general anesthesia, before and during TAVR, to further clarify LVOT morphology and valve implantation depth; (4) all patients who required monitoring for 30 minutes after TAVR, and DSA and for whom TEE findings were used in combination to confirm that there was no valve displacement; (5) patients who were generally stable and successfully discharged after transfer to the general cardiology ward; and (6) all patients who underwent TAVR via femoral access under general anesthesia using a new-generation self-expanding valve made in China (VitaFlow LibertyTM, MicroPort, Shanghai, China).

Study exclusion criteria were: (1) patients with a peak aortic valve pressure gradient over 20 mmHg, as measured by TTE before TAVR; (2) patients with failed surgical bioprosthetic heart valves; (3) patients in whom TAVR failed because the valve stent could not be anchored; (4) patients with a preoperatively fitted pacemaker; (5) patients who received non-VitaFlow Liberty^TM^ valves (Microport, Shanghai, China); (6) patients who were directly transferred intraoperatively for surgery; and (7) patients who died from complications before discharge or were in poor health at discharge.

[1] Vahanian A, Beyersdorf F, Praz F, Milojevic M, Baldus S, Bauersachs J, et al. 2021 ESC/EACTS Guidelines for the management of valvular heart disease. Eur Heart J. 2022; 43: 561–632. https://doi.org/10.1093/eurheartj/ehab395.

[2] Writing Committee Members, Otto CM, Nishimura RA, Bonow RO, Carabello BA, Erwin III JP, et al. 2020 ACC/AHA guideline for the management of patients with valvular heart disease: a report of the American College of Cardiology/American Heart Association Joint Committee on Clinical Practice Guidelines. J Am Coll Cardiol. 2021; 77: e25–e197. https://doi.org/10.1016/j.jacc.2020.11.018.

[3] Baumgartner H, Falk V, Bax JJ, De Bonis M, Hamm C, Holm PJ, et al. 2017 ESC/EACTS Guidelines for the management of valvular heart disease. Eur Heart J. 2017; 38: 2739–91. https://doi.org/10.1093/eurheartj/ehx391.
